# Instability in X chromosome inactivation patterns in AMD: a new risk factor?

**Published:** 2013

**Authors:** Bajic Vladan, Spremo-Potparevic Biljana, Vesna Mandusic, Milicevic Zorana, Lada Zivkovic

**Affiliations:** 1 Institute of Pharmaceutical Research and Development, University of Belgrade and Galenika a.d.; 2 Department of Physiology, Faculty of Pharmacy, University of Belgrade.; 3 Institute for Nuclear Sciences’’ Vinca’’, Department of Molecular Biology and Endocrinology, Belgrade, Serbia.

**Keywords:** X Chromosome, AMD, DIAPH2 Gene, X Chromosome Skewing

## Abstract

Years ago, it was thought that a genetic component was the fundamental cause of a number retinopathy diseases including age related macular degeneration (AMD). Since then, information has emerged about novel genes that contribute to various forms of AMD and other retinopathies that have been eluding researchers for years. In the genetic sense, only the APOE 2 and 4 genes have been found to be a risk factor for sporadic AMD. But, a recent Genome wide association study (GWAS) revealed that an alteration of five SNIPs on the X chromosome in a gene named DIAPH2 may be a susceptibility gene for AMD. Furthermore, the gene DIAPH2 showed to have a polygenic pleiotropy for premature ovarian failure (POF) and AMD in a cohort of women. POF is highly associated with X chromosome skewing, an epigenetic alteration of the inactivation process of the X chromosome. These findings suggest a hypothesis that an epigenetic alteration on the inactivation centres of the X chromosome (or skewing) relates not only to aging, but might be a novel property that affects women with AMD more often than men.

## INTRODUCTION

The human X chromosome is unique and its uniqueness is based on the fact that 5% of the human genome is concentrated on the X chromosome and that the duplication of this genetic material in women demands systematic compensation of the gene dosage in order to silence one the copy of the X chromosome [1] . Age Related Macular degeneration (AMD) is the most prevalent form of retinopathy in the elderly. Furthermore, there is a slight prevalence of female susceptibility to AMD [2,3] suggesting the involvement of the X chromosome. In AMD, the X chromosome is not prevalent in comparison to other chromosomes [4] but as we shall encounter, a possible role in epigenetic control of X chromosome inactivation might be considered as a novel pathway in AMD pathogenesis. 

Two basic forms of AMD have been the focus of research [4-6]. Central geographic atrophy is referred to the "dry" form of advanced AMD. This form results from atrophy of the retinal pigment epithelial layer below the retina, which causes vision loss through loss of photoreceptors (rods and cones) in the central part of the eye. The other form is names "wet" or neovascular/exudative AMD which is related to abnormal vessel growth in the choriocapillaris through the Bruch’s membrane, leading to a leakage of protein and blood in the macula [5-6]. These two forms account for almost 2 million people in the US alone and seven million people at risk [6]. With today’s increased life expectancy, this devastating disease will continue to have a significant public health impact on the quality of life worldwide [6]. Epidemiological studies provide a conclusion of a strong interplay of epigenetic, genetic and environmental factors in AMD pathogenesis [7]. At present, aging [7,8], chronic oxidative stress [9], inflammation [10], vascular disease [11], UV exposure [12], nutritional status [13], altered fatty acid metabolism [14], are strongly linked to AMD [15] suggesting that AMD etiology is known to be multifactorial, i.e., in addition to a strong genetic component. Still, advanced age and family history are the two major risk factors [16]. To understand AMD in the context of aging we will address the X chromosome not to its genetic alterations but its unique epigenetic properties to manifest inactivation of one of its X chromosomes in women and also its relation to skewing which may also affect male subjects [17]. 

**X linked genetics in retinopathies and AMD**

**Figure 1:**
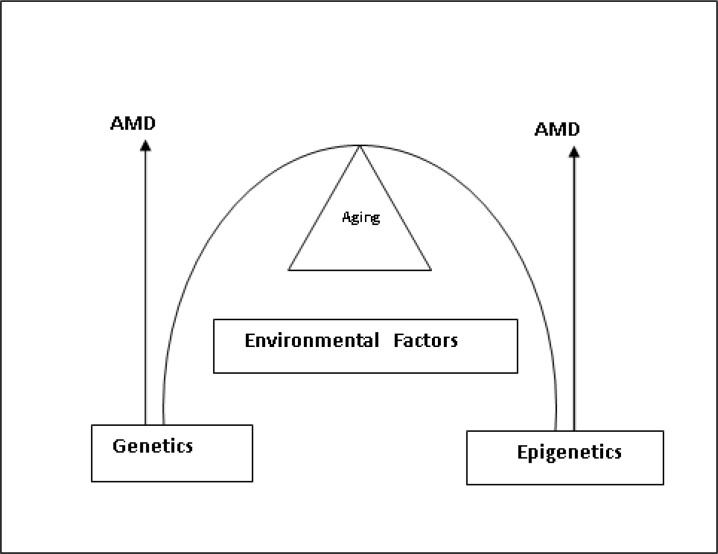
AMD is shown to be a consequence of a complex process of intertwining genetic, epigenetic and environmental factors with aging

The X chromosome harbors a number of disease loci that affect the retina, including cone dystrophy (COD1 and COD2), juvenile retinoschisis (XLRS), congenital stationary night blindness (CSNB), and at least five loci for retinitis pigmentosa (Table 1, ref [18, 19-35]). Still, a number of gens have been cloned and mapped that of macular degeneration, but not for the X chromosome, except some deletions in green and red color genes at Xq28 (Table 1) [34-35]. Families affected by these X chromosome deletions reveal progressive cone dystrophy and unusual cases with progressive macular atrophy in families with blue cone monochromacy (Table 1) [34-35]. The uniqueness of the X chromosome is addressed as not to its loci that harbor genetic changes linked to AMD (Table 1) but also a strong link to epigenetic mechanisms that may influence the genetic component in AMD (Figure 1).

**Epigenetics of AMD, relation to X chromosome?**

A number of retinopathy genes have been identified and characterized (Table 1) and also recent GWAS analysis has broadened our knowledge of the complexity of the disease [36]. Using GWAS as stated, recently 5 SNIPs are found related to susceptibility to AMD with a slight gender preference [3]. However, for many diseases, including AMD, the variation in phenotype with a single genotype, individual susceptibility to disease, different findings in monozygotic twins, polygenic pleitropy, its age-related onset cannot be explained only by acquiring mutations in genes or chromosome aberrations alone [37]. There must be another layer of information (Figure 1). This missing link could be epigenetic factors [37-38]. A vast spectrum of epigenetic changes has been described. The most common epigenetic variations involve DNA methylation, various modifications of histones, chromatin remodeling, microRNA (miRNA) and small non-coding RNA expression [37-38]. Epigenetics are involved in gene expression in eye development [38]. 

Epigenetic mechanisms influence gene expression and function without modification of the base sequence of DNA and may be reversible, heritable, and influenced by the environment [38-39]. Epigenetics are intertwined with environmental factors such as nutrition, smoking, pollution and may express genes that are not normally triggered, therefore increasing disease susceptibility, phenotypic variation and age related progression of many common diseases including AMD, glaucoma, Alzheimer’s disease [39]. Lin et al [40] revealed that inflammation that is associated with glycation end products (AGEs) stimulates inflammatory genes in RPE cells in vitro leading to the conclusion that there is not only a complex interplay of environmental factors with our genes but we must account our epigenome to the overall picture of AMD (Figure 1). 

**Table 1:** X chromosome linked Retinopathies

**Location of chromosome **	**Affected protein, diseases and comments**	**Ref.**
Xp22.2	Jobert syndrome; X-linked retinitis pigmentosa, severe; genomic next-generation sequencing also uncovered a deep intronic mutation in a family with severe X-linked RP (the RP23 locus)	[18]
Xp21.33	AMD and premature ovarian failure, near the DIAPH2 gene, which is known to cause premature ovarian failure (POF) in females	[3]
Xp22.13	Retinoschisis; protein: retinoschisin expression is limited to photoreceptors but protein is secreted into the inner retina	[19]
Xp21-q21	Retinitis pigmentosa with mental retardation	[20]
Xp21.3-p21.2/ Xp11.4	X-linked retinitis pigmentosa retinitis pigmentosa GTPase regulator	[21- 22]
Xp21.2-p21.1	Oregon eye disease, candidate gene; exons 20-28 involved in retinal disease	[23- 24]
Xp11.4	X-linked congenital stationary night blindness; protein: nyctalopin	[25- 26]
Xp11.3-p11.23	retinal dysplasia, primary linked to the Norrie disease	[27]
Xp11.23	X-linked progressive cone-rod dystrophy; protein: L-type voltage-gated calcium channel alpha-1 subunit	[28]
Xp11.23	protein: retinitis pigmentosa 2 (X-linked)	[29]
Xq21.1	Retinitis pigmentosa with myopathy; protein: phosphoglycerate kinase	[30]
Xq22.1	optic atrophy, protein: inner mitochondrial membrane translocase 8 homolog A., protein involved in transport of metabolites into mitochondria	[31]
Xq26-q27	X-linked retinitis pigmentosa	[32]
Xq27	X-linked progressive cone dystrophy, genetic heterogeneity underlying this disease entity	[33]
Xq28-qter	X-linked retinitis pigmentosa	[34]
Xq28/Xq28	Rare macular dystrophy in blue and red cone monochromacy with loss of locus control element; protein: green cone opsin and red cone opsin	[35]

* X chromosome ideogram based on G-bands with approximate location of the centromere region

A good example is found in twin studies. Seddon et al reported a difference between two twins [41]. One twin has been found to have a large drusen and pigmentation area accompanied with an advanced stage of AMD and these findings where related to heavy smoking. The other twin had smaller drusen size and area, and less pigmented. In his nutrition he tended to have higher dietary vitamin D or methionine intake. These findings suggest factors that are associated with epigenetic mechanisms are involved in the etiology of AMD, in addition to genetic susceptibility [40-42]. These epigenetic changes may be age-related and cell or tissue specific. They may also persist throughout the lifetime of an individual. An understanding of the role of epigenetics is important to the success of the stem cell-based therapies [42-44]. Our hypothesis is that the X chromosome has a more important role than it is noted today, in an epigenetic way. By the notion that the same genes that are responsible for genetic inheritance for AMD are also stimulated by an appropriate environmental factor that relates to AGEs and that inflammation is a key factor to AMD susceptibility may show a mechanistic pathway of how genes on the X chromosome may be influenced by alterations of X chromosome inactivation patterns [40,43-44].

## HYPOTHESIS

We postulate a hypothesis of an epigenetic influence on the inactivation patterns of chromosome X in the pathogenesis of AMD. These changes of the inactivation patterns are related to skewing. Skewing by definition is a non-random X-inactivation process.

**Evaluation and Discussion of the Hypothesis:**

**Instability in X chromosome inactivation patterns: X chromosome skewing**

It has been hypothesized by Lyon in 1961 [45] that one the pair of X chromosomes in women is always inactivated in early embryonic stage. It is quite clear that; inactivation of the X chromosome is a complex epigenetic process. This mechanism keeps one individual transcriptional active chromosome per cell. In an YXX embryo the active chromosome is always the mother’s chromosome where the father is always inactivated. By the time of implementation of the egg, inactivation is erased and re-instated on the basis of randomness in the embryo, except for the germinate line where both X chromosomes are inactivated and in the placenta where only the mother’s X chromosome is expressed. Therefore, X chromosome inactivation is a random process and it is stably inherited through cell cycling which means women universally differ as to which chromosome is inactivated. Skewed X chromosome inactivation (XCI) is a marked deviation from a 50:50 ratio and is arbitrary defined, often as preferential inactivation of either maternally or paternally inherited X chromosome in 75- 80% or more of cells [46].

Skewed X inactivation of one the X chromosomes can indicate that there exists some mutations on genes on the inactive X chromosome. So, here on the X chromosome recessively or dominant inheritance is relative because heterozygous women can express a phenotype which can be compared to women’s phenotypes as a result of X chromosome inactivation [46]. Inactive X chromosome acquires a number of features of heterochromatin such as hypermetylation, hyper condensation, late replication pattern, depletion of acetylated histons [46-47]. These characteristics enables identification of the inactive X chromosome on the cytogenetic level (late replication pattern analysis, analysis of Barr body) and molecular level most analyzed by using methylation patterns [47,48]. This difference in the sensitivity to methylation can be used to analyze non-random inactivation processes in diseases that are X chromosome linked, determination of tumor clonality and iPSC adaptability, i.e., iPSC cells show that skewing is not random. Initially balanced populations consistently skewed toward a “dominant subpopulation,” expressing genes from a dominant X allele [43-44]. These cells proved to be more efficient in reprogramming, more adaptable, than the non-dominant populations. These findings indicate that differences in X-linked-associated gene function and/or expression levels might influence a cell’s ability to deal with various environmental stresses, such as DNA damage, and/or affect its replicative life span. 

**A skewed view of X chromosome inactivation in AMD**

A recent new study of the involvement of the X chromosome in AMD has been suggested by Jiang et al [3] using a simple test for haplotype analysis of the X chromosome. This test applies haplotype-sharing (HS) statistics with sliding windows for males and females separately, which are then combined to a single HS test for the X chromosome. It should be noted that the haploid test is more powerful than single marker analysis [36]. The HS test was applied to genome wide association study GWAS of the National Eye Institute: Age Relate Eye Study NEI AREDS using 1804 SNPs [3]. What are interesting issues in this study are the results that have connected premature ovarian failure (POF) with AMD. POF is known to have an alteration of inactivation process of the X chromosome (skewing). Previous GWAS study [7] analyzed 100000 SNPs detected associations. Moreover, using the same subset of SNPs, Zheng et al. [36] conducted a single marker analysis on 1804 SNPs. Jiang et al [3] used a slight different strategy for analysis of the X chromosome in GWAS. First they used single maker analysis to rank SNPs on the X chromosome and the top ranked have been selected for haplotype analysis. This haplotype analysis from the GWAS studies confirmed risk associations for AMD as former results using single markers. Moreover, a chromosomal region consisting of five SNIPs has been associated to AMD [36]. The new research has found a disease preventive haplotype (ATGAC) in this region of SNIPs. These five SNPs are covered by the gene DIAPH2, which is known to cause premature ovarian failure (POF) in females. To date no relation between POF and AMD has been reported, although some relations have been noticed between AMD and POF [49-50]. Even though, Bretheric et al [51] by using FISH and PCR didn’t find any potential regions of DNA addition or deletion in the X chromosome, he still suggests that X-chromosome abnormalities undetectable by the arrays, or reduced follicular pool due to an early trisomic rescue event, may explain the skewed XCI observed in POF patients with primary amenorrhea. With the work of Baronchelli et al [52] these results suggest that a strong genetic factor in the etiology of POFis emphasized by the high percentage of familial cases in which X chromosome abnormalities account for 10% of chromosomal aberrations and that these alterations are possibly accompanied by epigenetic changes to the X chromosome inactivation patterns, or X chromosome skewing. Better said, numerous cases of POF in women with X-chromosome deletions or translocations have been reported; thus, it is possible that smaller rearrangements undetectable by conventional cytogenetics may contribute to cells with inactivation of the normal X chromosome and may be selected against, causing skewed X-chromosome inactivation (XCI) in AMD.

**Aging and skewing**

X chromosome skewing is affected by the aging process. This age-related, acquired XCI skewing can lead to late-onset disease in individuals, as has been reported for X-linked sideroblastic anemia [53] however if age-related, acquired XCI skewing occurs in tissues other than blood, then late-onset symptoms might be a concern for additional X-linked disorders which includes a number of other diagnosis such as premature ovarian failure, scleroderma (an autoimmune disorder), spontaneous abortions, a number of cancers, most often breast cancer [54]. In some severe X-linked disorders, post-inactivation selection takes place against the X chromosome carrying the mutant allele, leading to a completely skewed X-inactivation pattern and may indicate an effect of X-linked genes on the development of these conditions [46,55-56]. What is interesting except X chromosome skewing is that no causative X-linked locus has yet been identified in such associations, so causes of XCI skewing in addition to selection needs to be considered. One view supports the model of increased skewing with age as a consequence of hematopoietic stem cell senescence [57]. The second view suggests age-related skewing is a combination of stochastic and genetic events [58]. 

All this suggests that the process of X inactivation and the resultant degree of skewing is clearly important for the expression of diseases with a strong genetic background, such as AMD. 

## CONCLUSION

The complexity of disease as in AMD shows that no one factor can be promoted to the causality of the disease, but instead we have to relate to the interplay of epigenetic and genetic processes intertwined by environmental factors as seen in Figure 1. The X chromosome, even though not solely the only intrinsic factor in the genetic sense to contribute to AMD, but its X-linked genes on the two X chromosomes summons such a genetic variation pool in which single nucleotide polymorphisms(SNPs) and deletions are very common and may affect various cellular functions [59]. For example, polymorphisms in promoter/enhancer sequences might influence gene regulation, whereas other SNPs may affect RNA splicing or protein function. The findings of five SNPs in in the DIAPH 2 gene susceptible to AMD [3] which is also the gene responsible of form of POF. POF is known to promote X chromosome skewing [52,60-61]. Most of the X-linked genes are not involved in sex determination but in various other processes such as free radical scavenging, housekeeping, apoptosis, cell cycle regulation, tumor suppression [43-44]. All these processes may have another level of activity, good or bad, through the process of X chromosome inactivation [62]. Carrel et al [63] observed that 15 % of linked genes escape inactivation. Escapees are present on active and inactive X chromosomes. This shows an unsuspected degree of expression heterogeneity among females. X chromosome shows also a heritable variation in X-chromosome inactivation pattern in normal females [64]. There is genetic effect on the imprinting of X-chromosome inactivation in humans. Male offspring of females with skewed X-inactivation patterns were three times more likely to inherit alleles at loci that were located on the inactive X chromosome than the active X chromosome [17,64-65] .To broaden this notion, Plenge et al [66] explored skewed X chromosome inactivation and X linked mental retardation disorders (XLMR) and found that mutation carriers demonstrate a strong association with skewed X inactivation. The XLMR mutations are present on the preferentially inactive X chromosome in all 20 informative female subjects from these families, indicating that skewing is due to selection against those cells in which the XLMR mutation is on the active X chromosome [66].

To our view, the hypothesis of X chromosome instability in AMD seen as skewing may be broadened by the view that we may have a detrimental effect to DNA repair mechanisms in aged subjects by processes of skewing and aging. Aging is known to decrease repair of DNA damage in the nuclei and in mitochondria [67-68]. So skewing and aging may determine the amount of accumulated cellular DNA damage. In correspondence to Figure 1, DNA damage depends also on environmental and/or genetic conditions. These conditions help or undermine the cells ability to respond to, and repair damaged DNA and to repair telomere shortening. Pomp et al. [44] reported that skewing in a heterogeneous population of iPSC cells can be alleviated by ectopic expression of telomerase, and that this process profoundly cooperates in DNA repair. Another more controversial theme is transgenerational epigenetic inheritance [69-71]. That is, the environment can stably influence the establishment of the epigenome. Together with the fact that there is X inactivation inheritance pattern suggests that an environmental event in one generation could affect the phenotype in subsequent generations. Whether there is any merit to these assumptions that X chromosome skewing maybe also transgenerational inherited and contribute to the cause of the complex disease such as AMD is an important question to be answered in future research.

## DISCLOSURE

Conflicts of Interest: None declared.
